# Identification of thioredoxin domain containing family members' expression pattern and prognostic value in diffuse gliomas via in silico analysis

**DOI:** 10.1002/cam4.5169

**Published:** 2022-09-15

**Authors:** Begüm Kocatürk

**Affiliations:** ^1^ Department of Basic Oncology Hacettepe University Cancer Institute Ankara Turkey

**Keywords:** bioinformatics, cancer biology, molecular biology, oncogenes, prognostic factor

## Abstract

**Background:**

Gliomas are the most prevalent primary tumors of the central nervous system. Their aggressive nature and the obstacles arising during therapy highlights the importance of finding new prognostic markers and therapy targets for gliomas. TXNDC genes are members of the thioredoxin superfamily and were shown to play a role in redox homeostasis, protein folding, electron transfer and also acting as cellular adapters. The well known contribution of these processes in cancer progression prompted us to investigate if TXNDC family members may also play a role in carcinogenesis, in particular diffuse gliomas.

**Methods:**

The present study used in silico analysis tools GEPIA, UCSC Xena, Gliovis, cBioPortal, and Ivy GAP to evaluate the expression pattern, prognostic value and clinical significance of TXNDC family members in diffuse gliomas.

**Results:**

Our analysis showed that TXNDC family members' expression pattern differ between tumors and healthy tissues and among tumors with different grades. The detailed analysis of TXNDC5 in glioma pathogenesis revealed that TXNDC5 expression is associated with more aggressive clinical and molecular features and poor therapy success both in LGG and GBM samples. Kaplan–Meier survival curves represented a worse prognosis for patients with leveated TXNDC5 levels in LGG and all grade glioma patients. The levels of TXNDC5 was shown to be possibly regulated by hypoxia‐ER stress axis and a potential mechanism for TXNDC5‐driven glioma progression was found to be extracellular matrix (ECM) production which is known to promote tumor aggressiveness.

**Conclusions:**

Our results uncovered the previously unknown role of TXNDC family members in glioma pathogenesis and showed that TXNDC5 levels could serve as a predictor of clinical outcome and therapy success and may very well be used for targeted therapy.

## Graphical abstract

Thioredoxin domain‐containing (TXNDC) family genes are potent antioxidants that also regulate protein folding. Among these genes, TXNDC5 is known to play a pro‐survival function under stress conditions which prompted us to investigate it further in cancer context. Our analysis revealed that TXNDC5 levels are elevated in glioma samples and its increment is correlated with an aggressive phenotype, low therapy success and poor prognosis. In addition, ER stress and hypoxia were found to be positive regulators of TXNDC5 expression which in turn promoting ECM‐related gene expression. Overall, TXNDC5 was determined to be a potential prognostic marker and target for therapy in glioma patients.

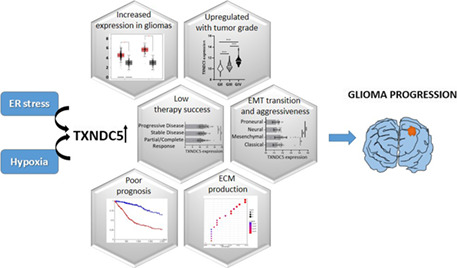

## 1 INTRODUCTION

The most common forms of primary tumors associated with the central nervous system are diffuse gliomas with their origins in glial cells. Standard therapy includes aggressive treatment regimens; however, their prognosis remains poor.^1^ Diffuse gliomas are classified into two major subtypes being low grade glioma (LGG, grade II/III) and glioblastoma multiforme (GBM, grade IV). LGG has a relatively better prognosis with a median survival rate varying between 5.6 to 13.3 years^2^ whereas GBM has a strong fatal phenotype with an overall survival time of approximately 14.6 months and 2 year survival rate of 26.5%.^3^ The invasive and therapy‐resistant nature of GBM plays a fundamental role in its poor prognosis. Interestingly, LGG has a high chance of transforming into secondary glioblastomas which is accompanied by changes in oncogenic pathways.^4^ Given these facts, it is clear that further studies investigating the regulatory mechanisms of glioma progression are valuable.

The close association between gene expression and cancer progression has been described in many studies.^5^ Thioredoxin domain‐containing (TXNDC) family genes are a part of the Protein Disulfide Isomerase (PDI) family and have 17 members. They play a crucial part in several mechanisms involved in cellular homeostasis and regulate proper protein folding by using their disulfide isomerase activity and by assisting electron transfer to other oxidoreductases.^6^ TXNDC proteins are also known to have antioxidative functions via downregulation of oxidative proteins.^7^, ^8^ Moreoever, they may act as cellular adapters.^9^ Given that perturbations in these processes are associated with malignant transformation, several previous studies have sought to understand their role in cancer progression. The first clue showing their involvement in cancer progression is based on the fact that their expression profile differs between tumor and normal tissues.^10^, ^11^, ^12^, ^13^ In hepatocellular carcinoma, higher TXNDC1, TCXNDC7, TXNDC9 transcript levels were shown to be associated with poor prognosis.^14^ It has been demonstrated that TXNDC9 promoted prostate cancer cell survival and proliferation.^11^ Higher TXNDC9 levels were also shown to be correlated with colorectal cancer progression and poor prognosis.^12^ In addition, TXNDC17 was found to be involved in chemotherapy resistance and associated with poor prognosis in ovarian cancer.^13^ TXNDC5, has been seen to be expressed in lung, prostate, colon, liver, gastro, ovary, breast, and renal cell cancer and has elevated levels in tumor tissues compared to healthy counterparts.^10^, ^15^ In lung and gastric cancer high TXNDC5 levels were shown to be correlated with poor prognosis^10^, ^15^ and TXNDC5 was reported to be associated with cell proliferation, migration, invasion, and therapy resistance in renal cell carcinoma.^15^ Strikingly, previous studies also attained a pro‐survival function to TXNDC5 under stress conditions.^16^

The reports investigating the role of TXNDC family members in cancer propagation, in particular difusse gliomas are very limited if not missing. We therefore used GEPIA, UCSC Xena, Gliovis, cBioPortal, and Ivy GAP online databases to unveil the expression pattern, clinical and prognostic value of TXNDC family members, in particular TXNDC5, in diffuse glioma progression. The results showed that the expression pattern of TXNDC family members differs between tumor and normal tissues in diffuse gliomas. TXNDC5 levels were found to be elevated with increased tumor grade, tumor aggressiveness and therapy resistance. Furthermore, we demonstrated that hypoxia‐ER (Endoplasmic Reticulum) stress axis is a likely mechanism controlling TXNDC5 expression. Moreover, TXNDC5 transcript levels was an independent prognostic factor of overall survival (OS), disease free survival (DFS), and progression free interval (PFI) in LGG and all grade gliomas. We also found evidence that ECM (extracellular matrix) production/organization is a likely mechanism for TXNDC5‐driven tumor propagation.

Overall, we have confirmed that TXNDC5 plays a pivotal role in malignant progression of glioma. Hence it may serve as a new prognostic marker or therapeutic target for diffuse gliomas. Currently there is no treatment targeting TXNDC5 or other oxidoreductases in cancer therapy thus further studies are warranted.

## 2 METHOD

### 2.1 Gene expression analysis in glioma and normal tissues

The Gene Expression Profiling Interactive Analysis (GEPIA) platform (http://gepia.cancer‐pku.cn/) was used to analyze gene expression levels between tumor and normal samples.^17^ Expression levels in tumors were assessed using The Cancer Genome Atlas (TCGA) dataset whereas transcript levels in healthy tissue samples were obtained from the Genotype Tissue Expression (GTEx) project. Boxplots were generated automatically by the GEPIA portal. The cut‐off of *p* value and fold change were defined as 0.05 and 1.5, respectively.

### 2.2 Gene expression based on histological grade

Samples from the TCGA‐GBMLGG dataset were analyzed using the GlioVis (http://gliovis.bioinfo.cnio.es/) web application.^18^ TXNDC5 expression in patients having different histological grades was assessed and plotted as log_2_(norm_count+1). In total 226 patients with grade II, 244 patients with grade III and 150 patients with GBM (Grade IV) were included in the query.

### 2.3 TXNDC5 transcript level analysis based on glioma subtype, therapy outcome success, and clinical features

For glioma subtype analysis in LGGs, primary tumors in the TCGA‐LGG dataset were subdivided into three groups being astrocytoma, oligodendrioglioma, and oligoastrocytoma. The TXNDC5 transcript level in each subtype was plotted as log_2_(norm_count+1). The expression levels of TXNDC5 in GBM subtypes (proneural, neural, mesenchymal and classical) were also assessed similarly in primary tumors from the TCGA‐GBM dataset. To understand the relation between TXNDC5 levels and therapy outcome both primary and follow‐up treatment success was determined using primary tumor data obtained from the TCGA‐LGG dataset. Differential TXNDC5 expression in groups with different person neoplasm cancer status was verified using TCGA‐LGG, TCGA‐GBM, and TCGA‐GBMLGG samples. All of the aforementioned analysis was performed using the UCSC Xena web tool.^19^

TXNDC5 levels based on clinical recurrence was assessed using the TCGA, Firehose Legacy datasets for primary tumors in LGG, GBM, and all grade patients using cBioPortal.

### 2.4 TXNDC5 level assesment based on molecular subtype

The analysis showing the TXNDC5 expression levels based on IDH, MGMT, and 1p/19q status was performed with the GlioVis web tool. For lower grade glioma, glioblastoma, and all grade glioma TCGA‐LGG, TCGA‐GBM, and TCGA‐GBMLGG data were used, respectively.

### 2.5 The assessment of genes' prognostic values

Patient overall survival, disease‐specific survival, and Progression Free Interval Kaplan–Meier plots were created on the UCSC Xena browser using records of primary tumor samples obtained from the TCGA‐LGG, TCGA‐GBM, TCGA‐GBMLGG cohorts. Gene expression data were divided into two groups according to median.

### 2.6 TXNDC5 expression regulation analysis

The cBioPortal for Cancer Genomics (http://www.cbioportal.org) was used to analyze the correlation between TXNDC5 expression levels and hypoxia scores in primary tumors recorded on the TCGA‐LGG and TCGA‐GBM datasets.^20^, ^21^ Both heatmaps and correlation graphs were created automatically by the portal.

The correlation between ER stress gene signature and TXNDC5 expression was determined via the GEPIA platform. The gene set HALLMARK_UNFOLDED_PROTEIN_RESPONSE was used to represent ER stress activation and was obtained from the Gene set Enrichment Analysis (GSEA) Molecular Signatures database (https://www.gsea‐msigdb.org/gsea/msigdb/index.jsp).^22^, ^23^

### 2.7 Correlation analysis of TXDC5 with ECM and EMT‐related gene transcript levels

Correlation between TXNDC5 and EMT marker genes (Vimentin, Twist1, Snail1, YKL‐40) transcript levels were determined by retrieving gene expression RNAseq illuminaHiseq data on primary tumors in the TCGA‐GBM cohort from UCSC Xena. A log_2_(X + 1) transformed RSEM normalize count was depicted on both axes.

The correlations between TXNDC5 and ECM‐encoding gene transcript levels of tumors from the Repository of Molecular Brain Neoplasia Data (Rembrandt) dataset were obtained from the GlioVis portal. A log_2_(norm_count+1) was plotted on both axes.

### 2.8 TXNDC5 expression localization and GO enrichment analysis

The Ivy Glioblastoma Atlas Project (IVY GAP) (https://glioblastoma.alleninstitute.org/)^24^ was utilized to determine the location of TXNDC5 in glioblastoma samples. TXNDC5 expression within the microdisected anatomical portions was depicted on the graph.

GO enrichment analysis was performed with the GlioVis platform. Microarray data from the Rembrandt dataset was divided into two groups based on high or low TXNDC5 expression levels. Differentially expressed genes between two groups were selected with the following criteria: |logFC| ≥ 2 and *p* < 0.05. Subsequently, the enrichment analysis was performed on biological process, molecular function, and cell compartment.

### 2.9 Statistical analysis

Statistical Analysis was performed using GraphPad Prism 8.10. Comparisons between several groups were determined via Kruskal Wallis test followed by Dunn's post hoc test for multiple comparisons. Comparison between two groups was performed using Student's *t*‐test. All values are represented as the mean ± SD.

Kaplan–Meier curves were plotted using the UCSC Xena database. The difference between the curves was determined by Log‐rank test. Correlation analysis was performed by calculating Pearson's correlation coefficients. *p* < 0.05 denotes statistical significance.

## 3 RESULTS

### 3.1 TXNDC family members show differential expression profiles between tumor and normal brain tissues

**FIGURE 1:**
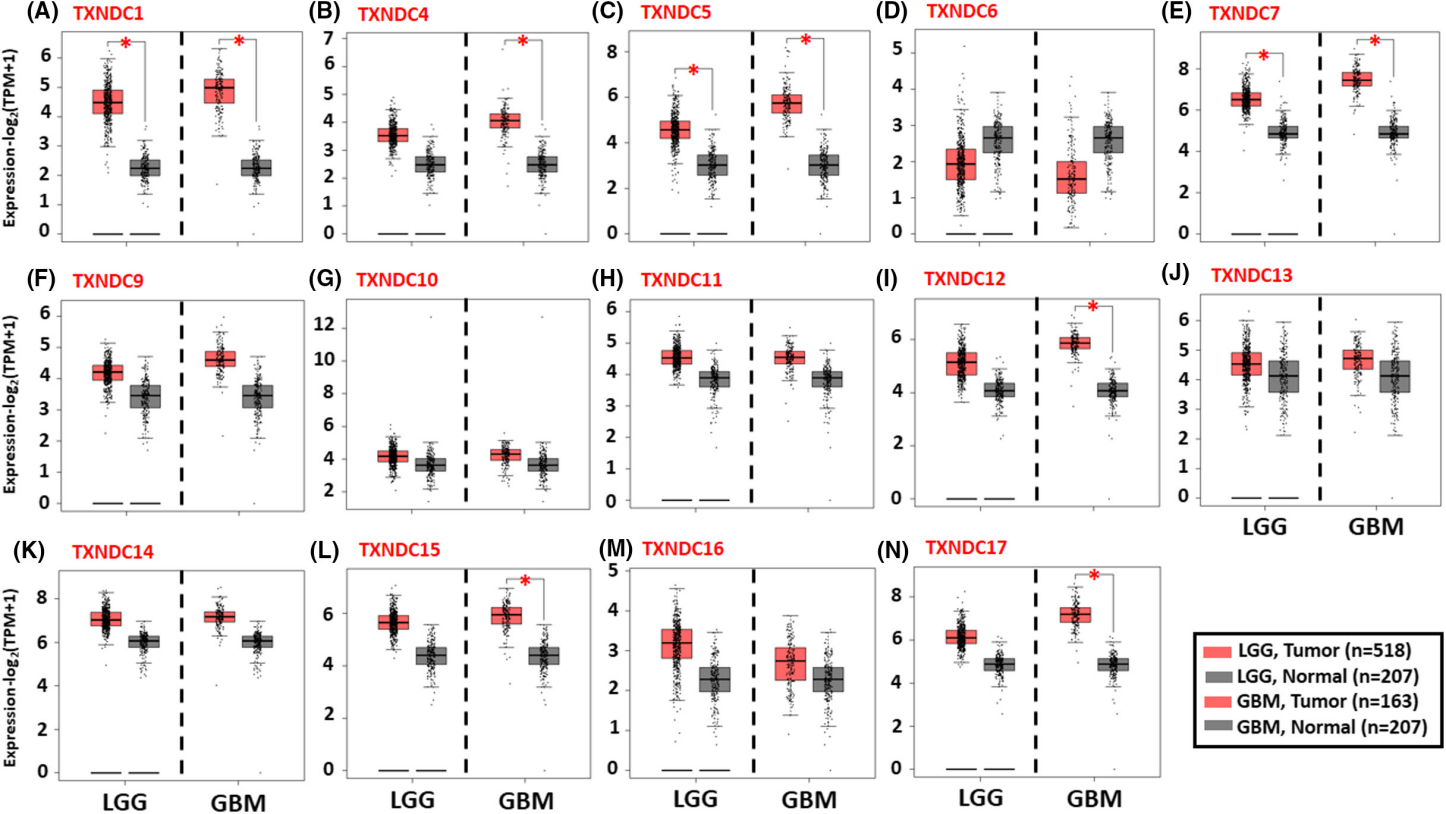
TXNDC family members show differential expression in LGM and GBM tumor tissues compared to normal counterparts. The transcript levels of TXNDC family members in LGG and GBM tumors and normal tissues were analyzed using GEPIA database. Box plots showing the expression profile of (A) TXNDC1, (B) TXNDC4, (C) TXNDC5, (D) TXNDC6, (E) TXNDC7, (F) TXNDC9, (G) TXNDC10, (H) TXNDC11, (I) TXNDC12, (J) TXNDC13, (K) TXNDC14, (L) TXNDC15, (M) TXNDC16, (N) TXNDC17 in tumor and healthy tissues. The asterisk (*) shows statistical significance (*p* < 0.05).

Cancer is known to be a complex disease which is enriched in modulations favoring its progression. Genes with a pro‐oncogenic nature have a tendency to be abundantly expressed whereas tumor suppressor gene levels show decrement in cancerous tissues.^5^ By using this straightforward approach, we aimed to understand if TXNDC family members show dissimilar expression in gliomas compared to normal brain tissues. The TXNDC family is composed of 17 members.^14^ However TXNDC2, TXNDC3, and TXNDC8 are exclusively expressed in testis^25^ thus throughout our paper, we focused on the analysis of the remaining 14 members. Our data revealed that TXNDC1 (Figure 1A), TXNDC5 (Figure 1C), and TXNDC7 (Figure 1E) transcript levels were significantly higher in all grade gliomas compared to healthy brain tissue. In addition, TXNDC4 (Figure 1B), TXNDC12 (Figure 1I), TXNDC15 (Figure 1L), and TXNDC17 (Figure 1N) transcript levels were upregulated in GBM samples compared to normal counterparts. Lastly, TXNDC6 (Figure 1D), TXNDC9 (Figure 1 F), TXNDC10 (Figure 1G), TXNDC11 (Figure 1H), TXNDC13 (Figure 1J), TXNDC14 (Figure 1K), and TXNDC16 (Figure 1M) did not show any changes in mRNA quantity between LGG, GBM, and healthy samples. Overall, these data underline a possible role for certain TXNDC family members in glioma progression.

### 3.2 TXNDC family members' expression pattern based on tumor grade and survival outcome

**FIGURE 2:**
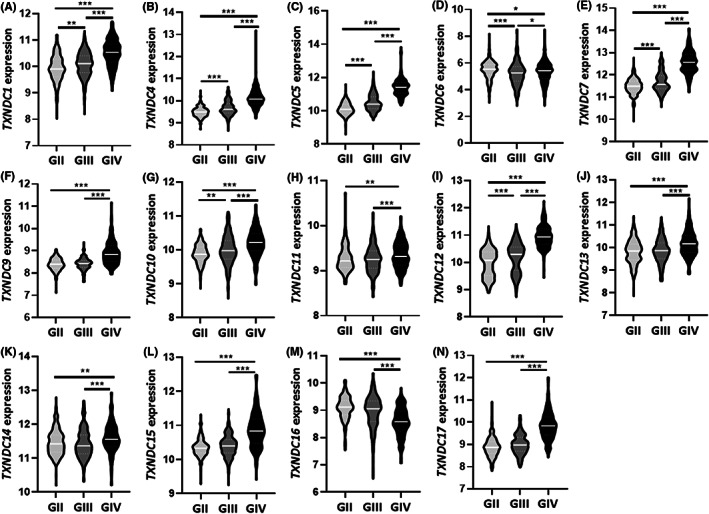
The expression characteristics of TXNDC family members among gliomas with different histological grades. GlioVis portal was used for the determination of the expression pattern of TXNDC family members in TCGA_GBMLGG dataset. Violin plots showing the transcript levels of (A) TXNDC1, (B) TXNDC4, (C) TXNDC5, (D) TXNDC6, (E) TXNDC7, (F) TXNDC9, (G) TXNDC10, (H) TXNDC11, (I) TXNDC12, (J) TXNDC13, (K) TXNDC14, (L) TXNDC15, (M) TXNDC16, (N) TXNDC17 in grade II, grade III and grade IV gliomas. The significance was set at *p* < 0.05 (*), *p* < 0.005 (**), *p* < 0.001 (***).

According to World Health Organization (WHO) diffuse gliomas are classified as lower grade glioma (LGG, grade II and grade III) and highly malignant glioblastoma multiforme (GBM, grade IV).^26^ To gain a better insight on the oncogenic nature of TXNDC family members, we analyzed their expression pattern based on tumor grade. Our result demonstrated that TXNDC1 (Figure 2A), TXNDC4 (Figure 2B), TXNDC5 (Figure 2C), TXNDC7 (Figure 2E), TXNDC10 (Figure 2G), and TXNDC12 (Figure 2I) levels showed a gradual increment based on tumor grade whereas TXNDC6 (Figure 2D), TXNDC9 (Figure 2F), TXNDC11 (Figure 2H), TXNDC13 (Figure 2J), TXNDC14 (Figure 2K), TXNDC15 (Figure 2L), TXNDC16 (Figure 2M), and TXNDC17 (Figure 2N) did not follow the aforementioned pattern. Altogether, the strong correlation between several TXNDC family members and tumor grade indicated that those members might very well regulate the malignant transformation of gliomas.

To unveil the likely prognostic value of TXNDC family members in glioma, we assessed their transcript levels in all grade glioma patients with different survival outcomes. We discovered that TXNDC1 (Figure S1A), TXNDC4 (Figure S1B), TXNDC5 (Figure S1C), TXNDC7 (Figure S1E), TXNDC9 (Figure S1F), TXNDC10 (Figure S1G), TXNDC12 (Figure S1I), TXNDC13 (Figure S1J), TXNDC15 (Figure S1L), TXNDC17 (Figure S1N) levels are significantly higher in patients who died rather than survive the disease. An opposite outcome was observed for TXNDC6 (Figure S1D) and TXNDC16 (Figure S1M) whereas there was no difference in transcript levels between living and deceased patients for TXNDC11 (Figure S1H) and TXNDC14 (Figure S1K). The strong association between TXNDC family members expression levels' and poor survival suggest a pivotial prognostic value for them and highlights the importance of detailed studies to understand their role in glioma progression.

### 3.3 TXNDC5 levels are elevated in astrocytomas and correlated with therapy success

So far, our analysis unveiled the previously unappreciated oncogenic roles of TXNDC family members in glioma. Among those members, we decided to proceed with TXNDC5 based on the following facts and observations: (i) TXNDC5 levels are higher in tumor tissues belonging to both LGG and GBM compared to healthy tissues (Figure 1C), (ii) TXNDC5 levels show positive correlation with diffuse glioma grade (Figure 2C), (iii) TXNDC5 levels are elevated in patients with poor survival characteristics (Figure S1C), (iv) TXNDC5 was shown to be involved in many disease pathogenesis including rheumatoid arthritis,^27^, ^28^ diabetes,^29^ and cancer,^30^ (v) there is no study investigating the role of TXNDC5 in glioma progression.

**FIGURE 3:**
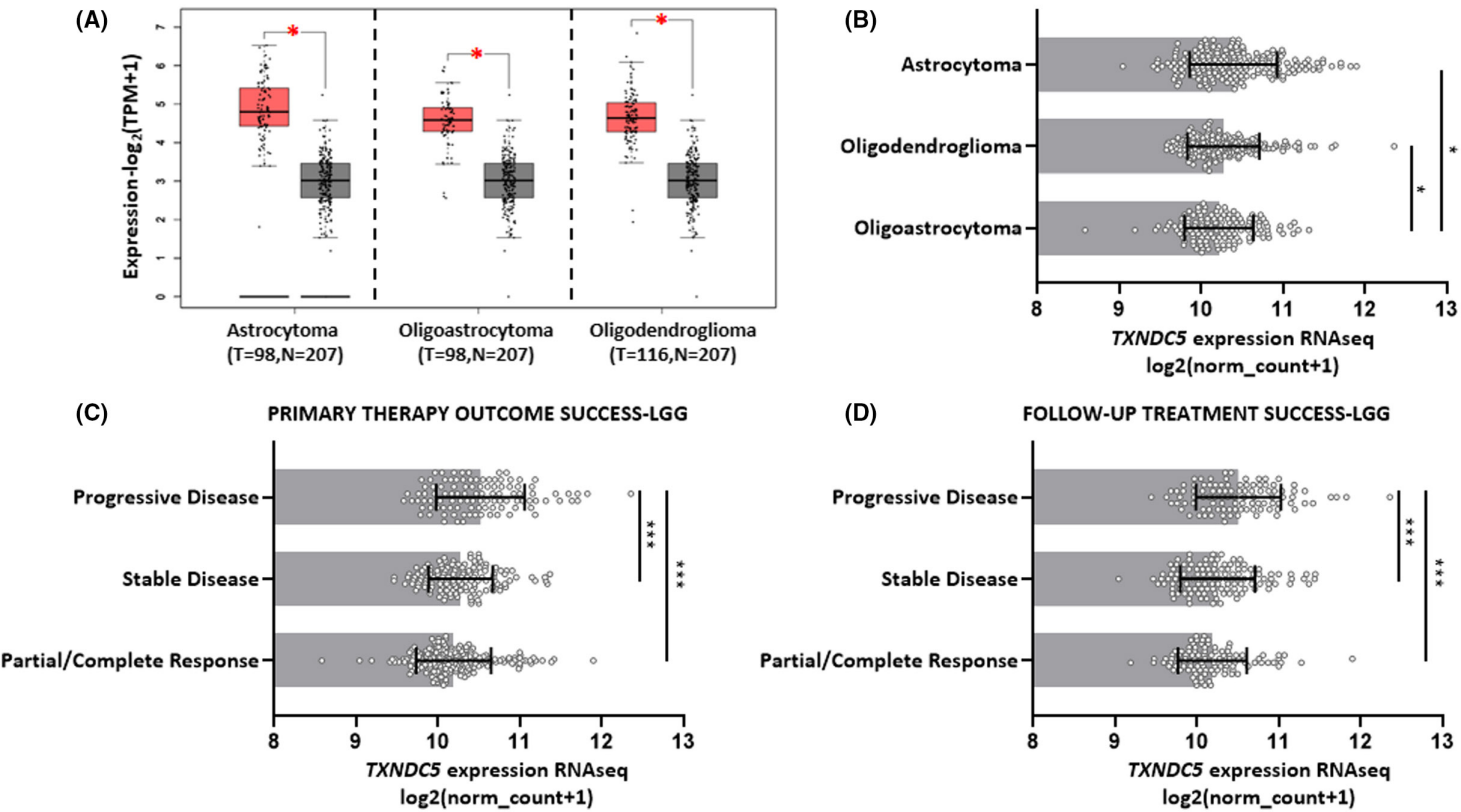
TXNDC5 expression levels according to LGG histological subtypes and response to therapy. (A) Boxplot showing TXNDC5 transcript levels in astrocytoma, oligoastrocytoma, oligodendrioglioma and corresponding normal tissues. The analysis was performed using TCGA_LGG and GTex dataset in GEPIA database. (B) Expression profile of TXNDC5 was compared among primary LGG tumor subtypes using UCSC Xena analysis tool. TXNDC5 transcript levels were plotted against (C) primary therapy outcome success and (D) Follow‐up treatment success of TCGA_LGG dataset via using UCSC Xena platform. The significance was set at *p* < 0.05 (*) and *p* < 0.001 (***).

Based on their histopathological characteristics, low grade gliomas are divided into three subtypes being astrocytoma, oligoastrocytoma, oligodendroglioma.^31^ In an attempt to understand the expression pattern of TXNDC5 better, we further analyzed its transcript levels in LGG subtypes. The transcript levels of TXNDC5 in all LGG subtypes are elevated compared to healthy counterparts (Figure 3A). In addition, TXNDC5 showed the highest expression in astrocytoma indicating that it might play a role in LGG progression, especially in astrocytomas (Figure 3B).

Surgery, radiation therapy, chemotherapy or a combined approach is used as a standard treatment regimen for LGG.^32^ Its treatment is crucial based on the fact that it can evolve into secondary GBM which carries a notorious therapy‐resistant nature^33^; hence, the treatment success is crucial for LGGs. In order to understand the possible contribution of TXNDC5 in this process, we investigated TXNDC5 levels in LGG patients having primary tumors. Our analysis showed that TXNDC5 levels are higher in non‐responders with progressive disease (Figure 3C,D).

### 3.4 TXNDC5 promotes the formation of an aggressive phenotype in GBM

**FIGURE 4:**
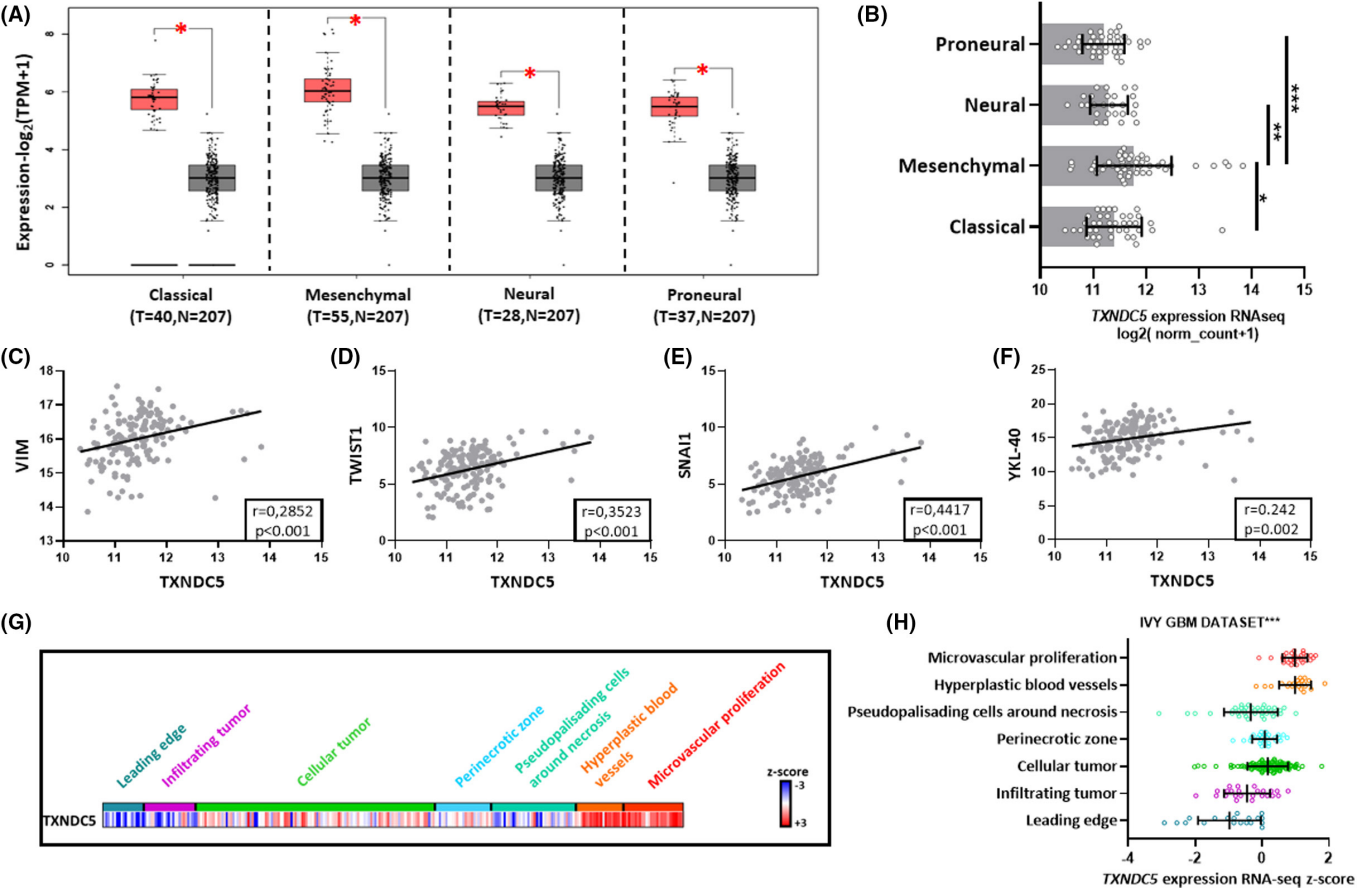
TXNDC5 plays a crucial role in GBM progression and outcome. (A) Analysis on TCGA_GBM database using GEPIA portal revealed that TXNDC5 expression levels in all GBM subtypes show marked increase compared to normal tissues. (B) Boxplot showing the distribution of TXNDC5 expression in different GBM subtypes based on TCGA_GBM dataset. Correlation of TXNDC5 levels with (C) VIM, (D) TWIST1, (E) SNAI1, (F) YKL‐40 in TCGA_GBM dataset. Pearson test was used for correlation analysis. (G) Heatmap showing the TXNDC5 expression profile based on its location in GBM samples. (H) TXNDC5 gene expression analysis in different locations of tumors showed increased TXNDC5 levels in hyperplastic blood vessels and microvascular proliferation zone. Data is obtained from The Ivy Glioblastoma Atlas Project (IVY GAP). The significance was set at *p* < 0.05 (*) and *p* < 0.001 (***).

Next, we also aimed to determine the expression pattern of TXNDC5 in histological GBM subtypes being classical, mesenchymal, neural, and proneural. We discovered that TXNDC5 transcript levels in all GBM subtypes are higher compared to normal brain tissues (Figure 4A). In addition, TXNDC5 was shown to be enriched in the mesenchymal subtype (Figure 4B) which is known to have increased aggressiveness and shows association with unfavorable outcomes.^34^, ^35^ This observation prompted us to hypothesize that TXNDC5 might induce epithelial to mesenchymal transition (EMT) activation. To verify that, we investigated if TXNDC5 levels show correlation with well‐known EMT markers' expressions.^36^, ^37^ Indeed, our analysis demonstrated that TXNDC5 expression positively correlated with Vimentin (Figure 4C), Twist 1 (Figure 4D), Snail1 (Figure 4E), and YKL‐40 (Figure 4F) transcript levels.

We further analyzed the transcription pattern of TXNDC5 in GBM samples based on tumor anatomic structure. Our data clearly demonstrated that TXNDC5 was enriched in the tumor area of hyperplastic blood vessels and microvascular proliferation (Figure 4G,H) which are known to contain highly proliferative cells and play a crucial role in tumor progression.^38^, ^39^

### 3.5 TXNDC5 expression is related to aggressive molecular glioma subtypes and clinical features

**FIGURE 5:**
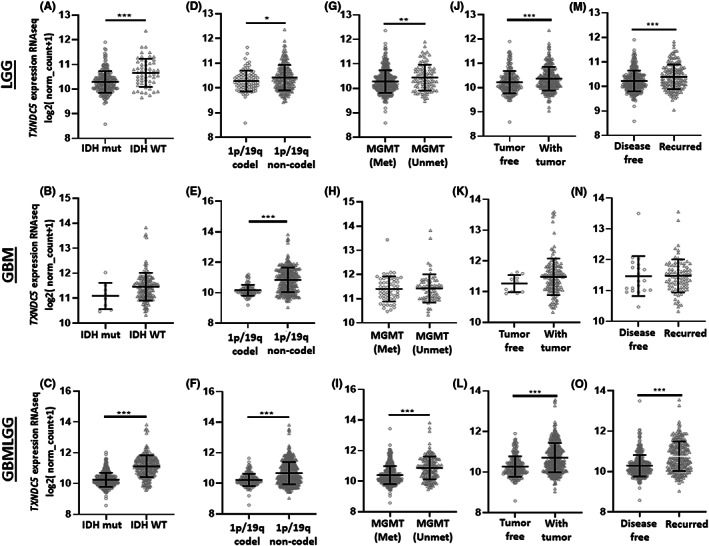
The expression pattern of TXNDC5 based on glioma molecular subtypes and clinical features. Differential expression of TXNDC5 between IDH mut and IDH WT tumors in (A) LGG, (B) GBM, and (C) all grade patients. Graphs representing TXNDC5 transcript levels between Chr1p/19q codeleted and non‐codeleted samples in (D) LGG, (E) GBM, and (F) all grade patients. Relative TXNDC5 levels in (G) LGG, (H) GBM, and (I) all grade patients based on MGMT status. TXNDC5 expression profile based on person neoplasm cancer status of (J) LGG, (K) GBM, and (L) all grade samples. Comparison of TXNDC5 transcript levels in disease free and recurred samples of (M) LGG, (N) GBM, and (O) all grade patients. All analysis were conducted on TCGA datasets. TXNDC5 levels in different molecular subtypes were assessed using Gliovis whereas expression analysis based on clinical outcome was determined via UCSC Xena and cBioPortal databases. The significance was set at *p* < 0.05 (*), *p* < 0.005 (**), *p* < 0.001 (***).

Our previous subtype analysis was based on histological characteristics. We also wanted to explore TXNDC5 levels in different molecular subtypes of gliomas. IDH mutation and 1p19q co‐deletion status are routinely screened in clinics and used as predictors for prognostic outcomes both in LGG and GBM^40^, ^41^, ^42^ whereas MGMT methylation status is rather used to assess the success of therapy.^43^ Our findings showed that LGG (Figure 5A) and all grade patients (Figure 5C) carrying IDH mutation exhibited elevated TXNDC5 expression compared to patients carrying WT IDH whereas there was no difference between these two groups in GBM (Figure 5B). Moreover, there is a clear upregulation of TXNDC5 trancript levels in patients with 1p/19q co‐deletion for all diffuse glioma types (Figure 5D–F). The groups lacking methylation on MGMT promoter have higher TXNDC5 levels in LGG (Figure 5G) and all grade patients (Figure 5I). However, the TXNDC5 expression did not differ between methylated and unmethylated groups in GBM patients (Figure 5H).

The progression status of the disease was evaluated using person neoplasm cancer status. Our analysis revealed that tumors with high progressive capacity have elevated levels of TXNDC5 in LGG (Figure 5J) and all grade glioma (Figure 5L) patients while TXNDC5 expression did not change with progressive phenotype in GBM group (Figure 5K). In addition, TXNDC5 levels were elevated in recurring LGG (Figure 5M) and all grade patients (Figure 5O) whereas there was no significant difference in TXNDC5 expression between disease free and recurred groups in GBM patients (Figure 5N). Altogether, TXNDC5 expression was found to be associated with aggressive tumor characteristics and therapy resistance which in turn may promote glioma propagation.

### 3.6 High TXNDC5 expression shows significant association with poor prognosis in glioma patients

**FIGURE 6:**
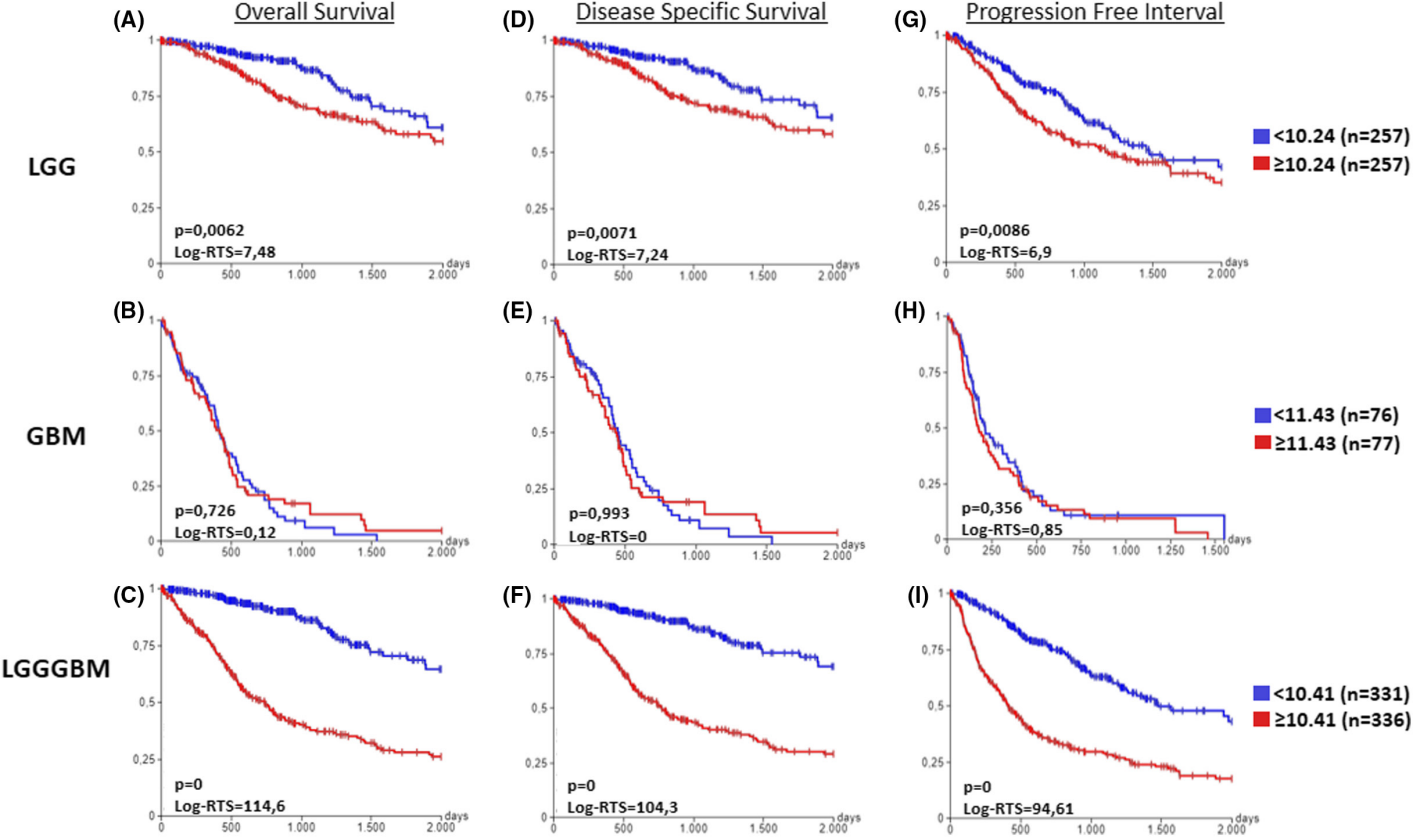
The prognostic value of TXNDC5 in glioma. Kaplan–Meier curves were created using UCSC Xena database and TCGA cohorts. Kaplan–Meier estimates of overall survival for (A) LGG, (B) GBM, (C) all grade patients. Kaplan–Meier estimates of disease specific survival for (D) LGG, (E) GBM, and (F) all grade patients. Kaplan–Meier estimates of progression free survival for (G) LGG, (H) GBM, and (I) all grade patients. The Log rank test was used to calculate the *p*‐value. *p* < 0.05 was considered statistically significant.

The data so far indicate that TXNDC5 may have a prognostic value in glioma patients. To validate the clinical relevance of TXNDC5, we conducted Kaplan–Meier analysis on primary tumors. Results showed that there is lower Overall Survival (OS) (Figure 6A), Disease‐Specific Survival (DSS) (Figure 6D), and Progression Free Interval (PFI) (Figure 6G) in LGG patients expressing high TXNDC5 than those expressing low TXNDC5. However, this does not hold true for GBM cases (Figure 6B,E,H). Last but not least, increased TXNDC5 levels in all grade glioma patients were strongly associated with poor OS (Figure 6C), DSS (Figure 6F) and PFI (Figure 6I).

### 3.7 TXNDC5 transcript levels' correlation with hypoxia and ER stress activation status

**FIGURE 7:**
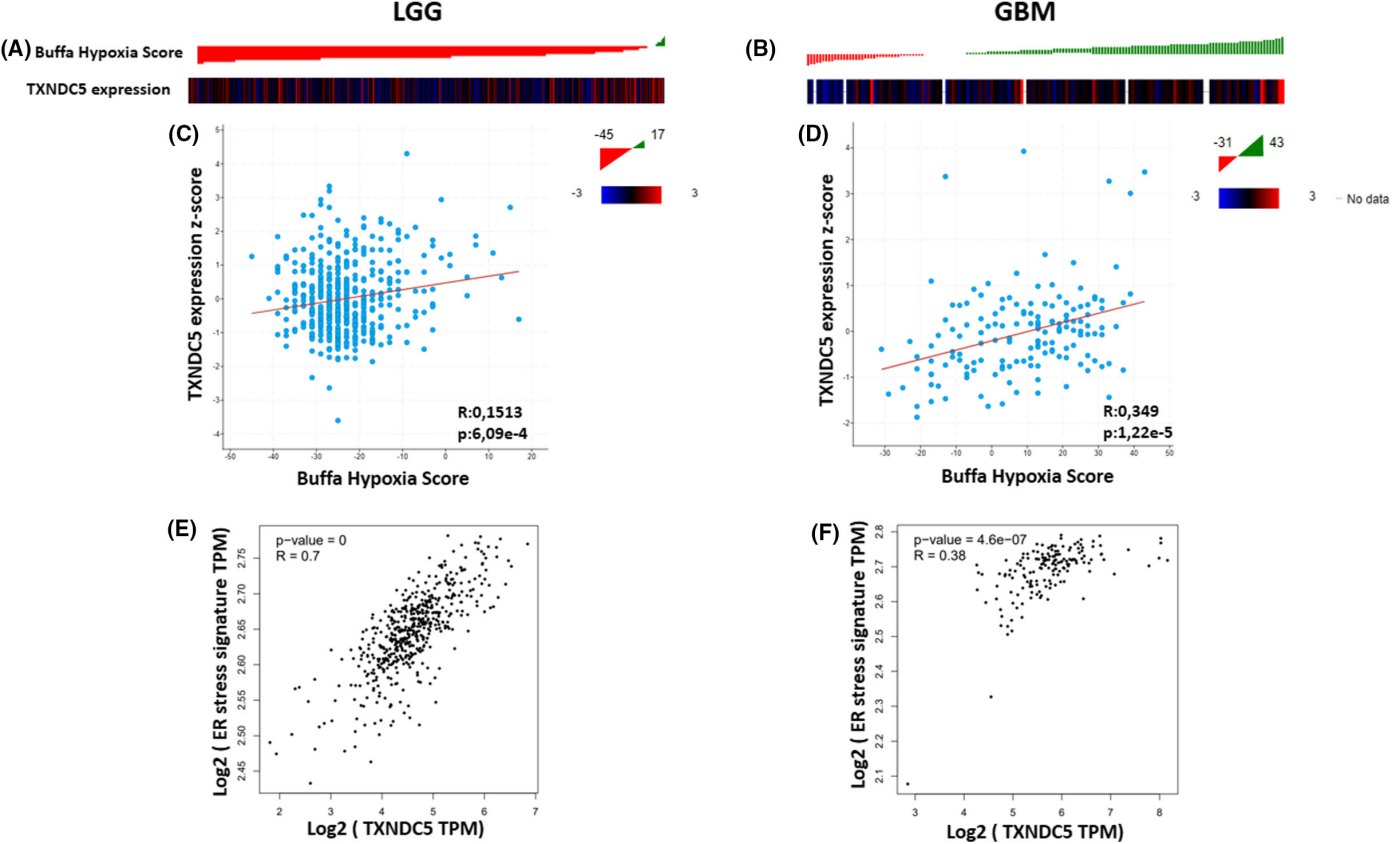
TXNDC5 expression is regulated by hypoxia and ER stress. Heatmap showing the TXNDC5 levels in (A) TCGA_LGG and (B) TCGA_GBM cohort based on buffa hypoxia score. Correlation analysis of TXNDC5 levels and Buffa hypoxia score in (C) TCGA_LGG and (D) TCGA_GBM using Pearson correlation test. The data is obtained using cBioPortal. Correlation analysis of expression of TXNDC5 and ER stress gene expression signature in (E) TCGA_LGG and (F) TCGA_GBM cohort via GEPIA database. *p* < 0.05 was considered statistically significant.

The increased TXNDC5 levels in gliomas prompted us to investigate the molecular mechanism regulating TXNDC5 expression. Previous studies have shown that in endothelial cells and the endothelium of tumors TXNDC5 levels are upregulated with hypoxia.^6^, ^44^ However, there is a lack of consistency on this observation and in non‐small cell lung carcinoma, TXNDC5 levels did not change with hypoxia.^45^ To clarify hypoxia's impact on TXNDC5 expression in a glioma microenvironment, we have performed a correlation analysis between tumor hypoxia scores and TXNDC5 transcript levels. Our data showed that TXNDC5 expression is positively correlated with the degree of hypoxia in GBM (Figure 7B,D) whereas there was a lack of correlation between hypoxia score and TXNDC5 levels in LGG (Figure 7A,C).

Upon perturbed homeostasis, ER activates a stress response which may result in the upregulation of specific genes to adjust the folding pattern of unfolded or misfolded proteins. The chaperone function of TXNDC5 makes it a strong candidate as an ER stress response gene hence making ER stress pathway a possible operator of TXNDC5 expression. Indeed, in clear cell renal cell carcinoma TXNDC5 levels were shown to be upregulated through this pathway^15^ and ATF6 seems to play a major role in this process.^46^ However, there are other reports also attaining a role for IRE1‐Xbp axis in TXNDC5 regulation.^47^ To verify the possible regulation of TXNDC5 by ER stress in all grade gliomas, we performed a correlation analysis. Our data showed a strong positive correlation between ER stress activation‐related genes and TXNDC5 levels in LGG (Figure 7E) and GBM (Figure 7F). Overall, our results identified hypoxia and ER stress as critical control points in TXNDC5 expression.

### 3.8 ECM‐encoding transcripts with prognostic significance shows positive correlation with TXNDC5 levels in diffuse gliomas

**FIGURE 8:**
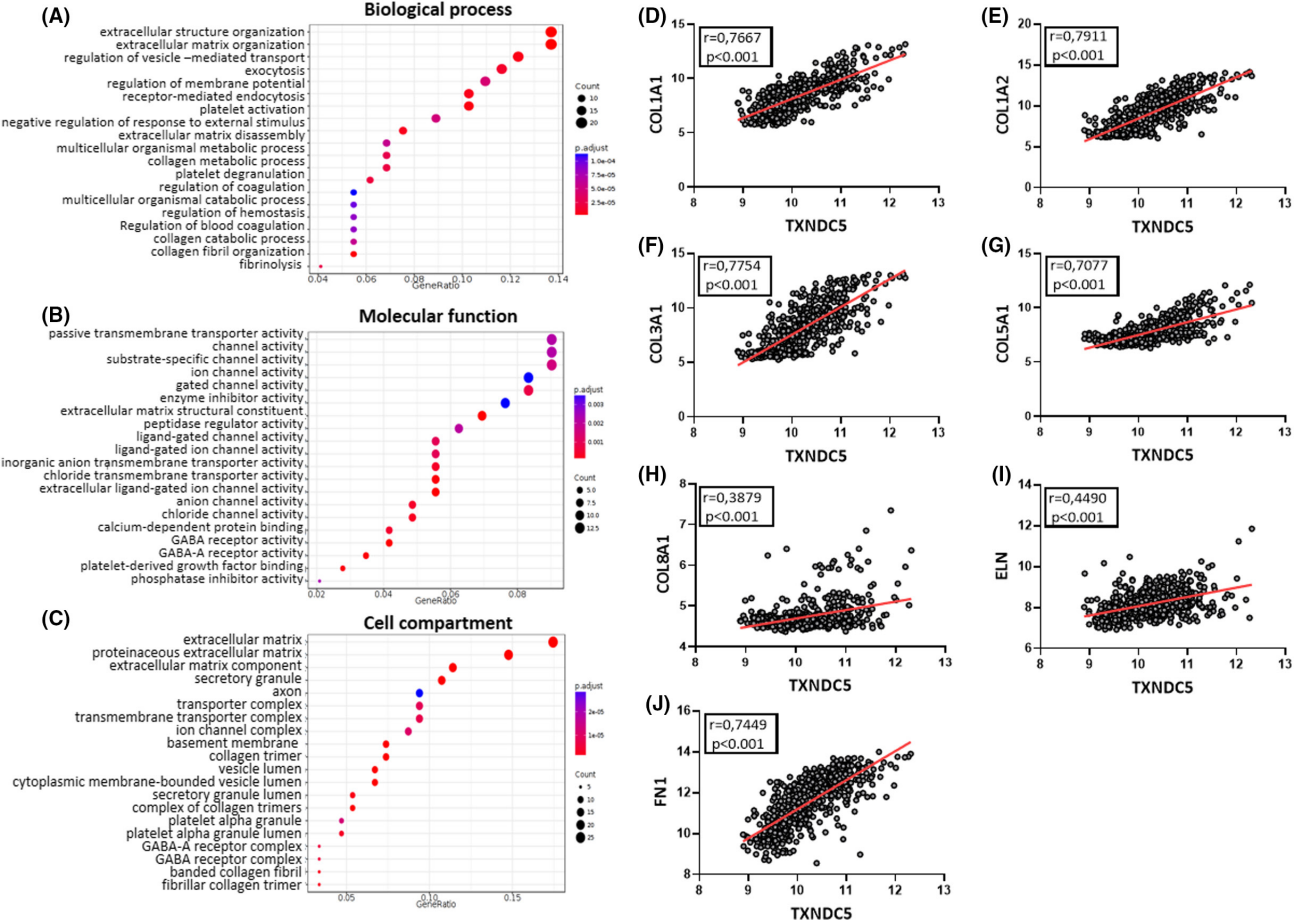
Enrichment analysis and ECM‐encoding gene transcript level correlation for TXNDC5. (A) Gene ontology biological process (B) molecular function and (C) cell compartment enrichment analysis for TXNDC5 gene were assesed using Gliovis database and Rembrandt dataset. Correlation of TXNDC5 levels with (D) COL1A1, (E) COL1A2, (F) COL3A1, (G) COL5A1, (H) COL8A1, (I) ELN, and (J) FN1 in Rembrandt dataset. Pearson test was used for correlation analysis. *p* < 0.05 was considered statistically significant.

To understand the possible mechanism lying behind TXNDC5‐directed glioma progression we performed a GO enrichment analysis (Figure 8A–C). The top terms appeared to be mostly related to ECM organization. Primary tumors of the central nervous system rarely metastasize to distant sites but rather have a tendency to invade locally and ECM is known to be a key player in this process.^48^ Previous studies showed that TXNDC5 and ECM‐encoding genes' transcript levels were correlated which indicates the presence of a possible control mechanism in the transcriptional level. In order to validate this finding in a glioma setting we have also performed a correlation analysis between TXNDC5 and the ECM‐encoding gene transcripts that are known to be involved in glioma progression. Our data clearly show a strong correlation between TXNDC5 and ECM‐encoding transcripts being COL1A1 (Figure 8D), COL1A2 (Figure 8E), COL3A1 (Figure 8F), COL5A1 (Figure 8G), COL8A1 (Figure 8H), ELN (Figure 8I), and FN1(Figure 8J).

ECM components are known to be involved in glioma progression^49^, ^50^, ^51^, ^52^ thus we wanted to further explore if the above‐mentioned ECM transcripts might also have a prognostic value in diffuse gliomas. Our Kaplan–Meier analysis demonstrated that high expression of COL1A1 (Figure S2A), COL1A2 (Figure S2B), COL3A1 (Figure S2C), COL5A1 (Figure S2D), COL8A1 (Figure S2E), ELN (Figure S2F), FN1 (Figure S2G) resulted in lower OS in all grade glioma patients, in turn causing poor prognosis.

## 4 DISCUSSION

TXNDC family member genes were shown to be associated with the progression of various cancers.^10^, ^11^, ^12^, ^13^ However, their clinical and prognostic values in glioma remain unclear. In the present study, we have uncovered the previously unknown role of TXNDC family members, in particular TXNDC5, in glioma progression. Herein, we showed that expression of several TXNDC gene family members were perturbed in tumor tissues compared to healthy counterparts implying their oncogenic role in diffuse gliomas. Moreover, their expression is further correlated with tumor grade and vital status underscoring their possible contribution to clinical outcome and prognosis through elevated levels of TXNDC5 in tumor tissues of both LGG and GBM patients, its gradual increase with the tumor grade, its strong elevation in deceased patients, and the presence of literature pinpointing its oncogenic nature^10^ encouraged us to investigate its role in glioma progression further.

Firstly, we evaluated the expression pattern of TXNDC5 in LGGs. Our results revealed that TXNDC5 levels were highest in the astrocytoma subtype. It is important to keep in mind that there is a high chance of low‐grade diffuse astrocytomas or anaplastic astrocytomas transforming into secondary GBMs with poor prognosis.^4^ The fact that TXNDC5 is expressed the most in this subtype might imply its involvement in this malignant transformation. In addition, relatively good prognosis of LGGs and the strikingly low overall survival rate of GBMs (~14.6 months) underlines the importance of therapy success for LGGs.^53^ The main therapy regimen used for gliomas is the combination of surgical resection, radiotherapy, and chemotherapy.^2^ Here we reported that the non‐responder LGG patients with progressive disease and patients with recurring disease have the highest TXNDC5 levels. Moreoever, a former study by Mo et al., showed that knockdown of TXNDC5 in renal cell carcinoma cells sensitizes them to chemotherapy.^15^ In line with these findings our analysis also showed that patients having a chemotherapy‐resistant profile accompanied by unmethylated MGMT promoter have increased TXNDC5 transcript levels. This aggressive phenotype was also recapitulated using molecular subtype analysis. LGGs with IDH mutation, and 1p/19q codeletion are known to have better prognosis.^2^ However, TXNDC5 expression was lower in these patients. Its prognostic value was also evaluated using Kaplan–Meier analysis and patients with higher TXNDC5 levels showed decreased OS, DSS, and PFI. Overall, our data clearly indicated that TXNDC5 is crucial for LGG progression, therapy outcome and has a prognostic value which in turn makes it a good candidate for targeted therapy.

We also analyzed the expression profile of TXNDC5 in GBMs. Among all the histological subtypes, TXNDC5 levels were highest in the mesenchymal tumors. This subtype is known to be the most aggressive with the worst prognosis.^54^ They originate either from the invasion of smooth muscle actin positive perivascular cells or via the the activation of mesenchymal transformation. A previous study by Wang et.al, reported that TXNDC5 is involved in EMT program in esophageal squamous cell carcinoma^55^ thus we have put our focus on the EMT pathway. Indeed, TXNDC5 levels showed strong correlation with mesenchymal markers verifying its role in EMT process for glioma as well. As GBMs are highly angiogenic, FDA approved the use of anti‐VEGF antibody bevacizumab for GBM patients^56^ indicating that anti‐angiogenic molecules might help clinicians to combat the disease. Our expression analysis based on anatomical location revealed that TXNDC5 is abundantly present in microvascular proliferation and the hyperplastic blood vessel zone. A former study showing the elevation of TXNDC5 levels in endothelial cells and endothelium of tumors via hypoxia might very well explain the increased TXNDC5 expression around vascular structures in GBM.^44^ Moreover, TXNDC5 was shown to promote angiogenesis.^57^ The involvement of TXNDC5 in pivotal tumor‐promoting pathways also prompted us to investigate its prognostic role in GBM. However, we could not detect any difference in OS, DSS, and PFI between TXNDC5 high and low groups. This might be due to the small number of GBM samples or different molecular mechanisms lying behind LGG and GBM progression. Altogether, we have shown that TXNDC5 induces GBM propagation via EMT and angiogenesis activation and might be a valuable therapy tool for GBM.

The altered TXNDC5 expression profile in tumors prompted us to further investigate the regulatory mechanism lying behind TXNDC5 expression. Previous studies showed that hypoxia induces TXNDC5 levels.^27^, ^44^, ^58^ We also evaluated the role of hypoxia in our setting and found a strong correlation between the hypoxia score and TXNDC5 levels in GBM tumors exclusively. This might be based on the fact that GBM has a significantly higher hypoxic nature compared to LGG (see hypoxia scores in Figure 7A vs. 7B) therefore hypoxia may not be the dominant regulator for TXNDC5 expression in LGG tumors and other regulatory mechanisms might be involved. Previous reports revealed that ER stress plays a fundemental role in the regulation of TXNDC5 expression and attained a role for IRE1^47^ and ATF6^59^ in TXNDC5 transcriptional regulation. Indeed, our analysis showed a significant correlation between ER stress signature gene expression and TXNDC5 levels implying that TXNDC5 is a part of ER stress response machinery. It is well known that upon perturbation of cellular homeostasis, ER activates pathways to rebalance the cellular homeostasis which promotes survival. Hypoxia induces oxidative stress, an important initiator of apoptosis.^60^ Previous studies demonstrated that TXNDC5 has antioxidative functions and downregulates oxidative proteins^7^, ^8^ thus blocking apoptosis. Moreover, protein folding is disturbed with hypoxia leading to accumulation of unfolded proteins.^61^ TXNDC5 was also shown to be involved in correct folding of proteins via its disulfide isomerase activity.^62^ The anti‐apoptotic nature of TXNDC5 was also recapitulated in the literature. Under hypoxic conditions, colorectal cancer cells showed increased apoptotic rates with TXNDC5 knockdown.^16^ Moreoever, under elevated ER stress conditions, NRD4A1 driven cell survival was shown to be mediated through TXNDC5 in a pancreatic cancer setting.^63^ Altogether, our data imply that TXNDC5 may help glioma progression via activation of anti‐apoptotic pathways under stress conditions.

Our GO enrichment analysis indicates additional mechanisms may be responsible for TXNDC5‐driven glioma propagation. We demonstrated that TXNDC5 was involved in extracellular matrix organization. Former studies showed the clear impact of TXNDC5 in ECM production. In a mouse model of pulmonary fibrosis, knockdown of TXNDC5 yielded lower expression of ECM protein genes,^61^ TXNDC5 was selectively enriched in collagen‐secreting fibroblasts in fibrotic mouse kidneys^64^ and TXNDC5 was also shown to be involved in ECM folding and production in cardiac fibrosis.^46^ Our data also revealed a strong correlation between ECM encoding genes and TXNDC5 levels. The increased ECM might promote cancer progression by acting as a shield around tumors, increasing hypoxia and metabolic stress thus diminishing therapy success.^65^ Hence, decreased therapy success with higher TXNDC5 levels might be due to increased ECM production. Moreover, ECM contributes to active migration and invasion of cancer cells^66^ and is involved in the blockade of immune surveillance^67^ that might explain the aggressive nature of gliomas with higher TXNDC5 levels. Previous studies showed an association between few ECM‐encoding genes and poor prognosis in glioma^68^, ^69^ which was also recapitulated in our study.

## 5 CONCLUSION

The present study demonstrated for the first time that TXNDC family members are differentially expressed in diffuse gliomas and in particular high TXNDC5 levels are associated with unfavorable clinopathological features and poor prognosis. Potential regulators of TXNDC5 expression were found to be hypoxia and ER stress pathways and higher TXNDC5 levels were shown to be associated with increased ECM production. Our findings suggested that TXNDC5 can be used as a therapy target and prognostic marker in glioma; however, further studies are warranted.

## AUTHORS' CONTRIBUTIONS

Begüm Kocatürk conceptualized the study, performed the analysis and wrote the manuscript.

## FUNDING INFORMATION

This study did not receive any funding from a particular source.

## CONFLICT OF INTEREST

The author has no competing interest to declare.

## ETHICS APPROVAL AND CONSENT TO PARTICIPATE

Not applicable.

## Supporting information


Figure 1

Figure 2

Appendix S1

## Data Availability

Raw data are available at GTEx, TCGA LGG, TCGA GBM cohorts and Rembrandt dataset. Further inquiries can be directed to the corresponding author/s.
