# Supplementary figures and images for: Identification of thioredoxin domain containing family members' expression pattern and prognostic value in diffuse gliomas via in silico analysis
Source: Cancer Med. 2022 Sep 15;12(3):3830–44. doi: 10.1002/cam4.5169 (PMC9939227; doi:10.1002/cam4.5169)

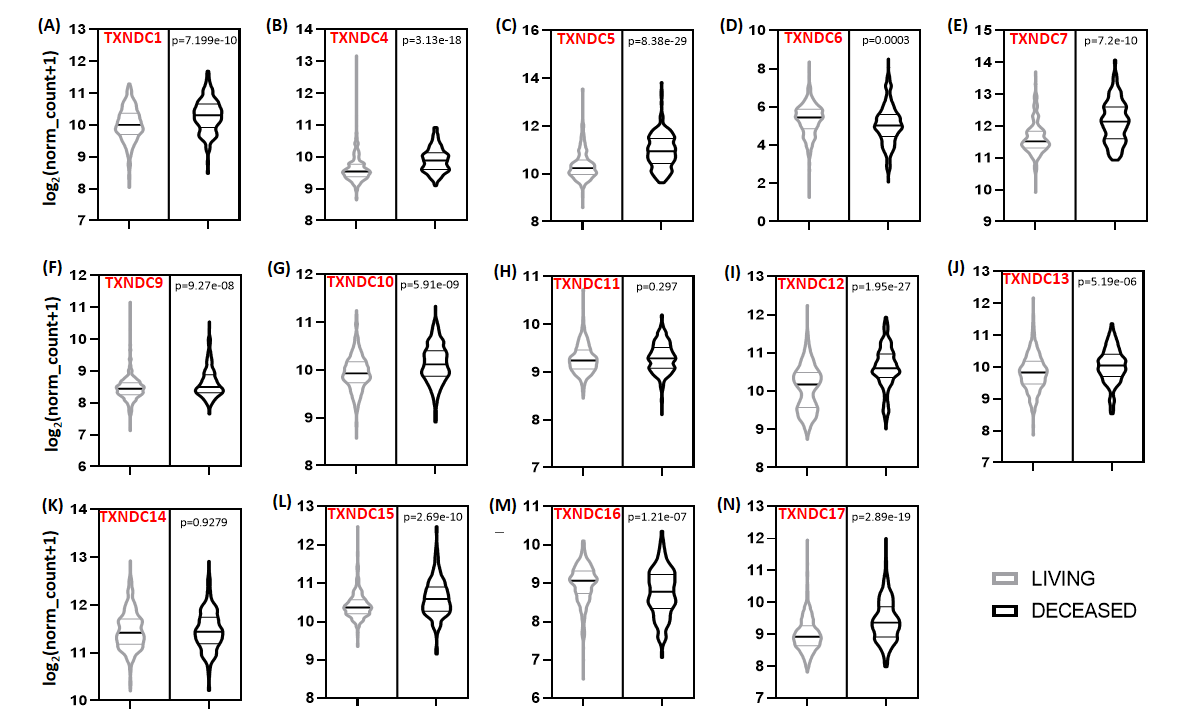

Supplement: Supplementary file 1 — Figure 1 [file CAM4-12-3830-s003.tif]

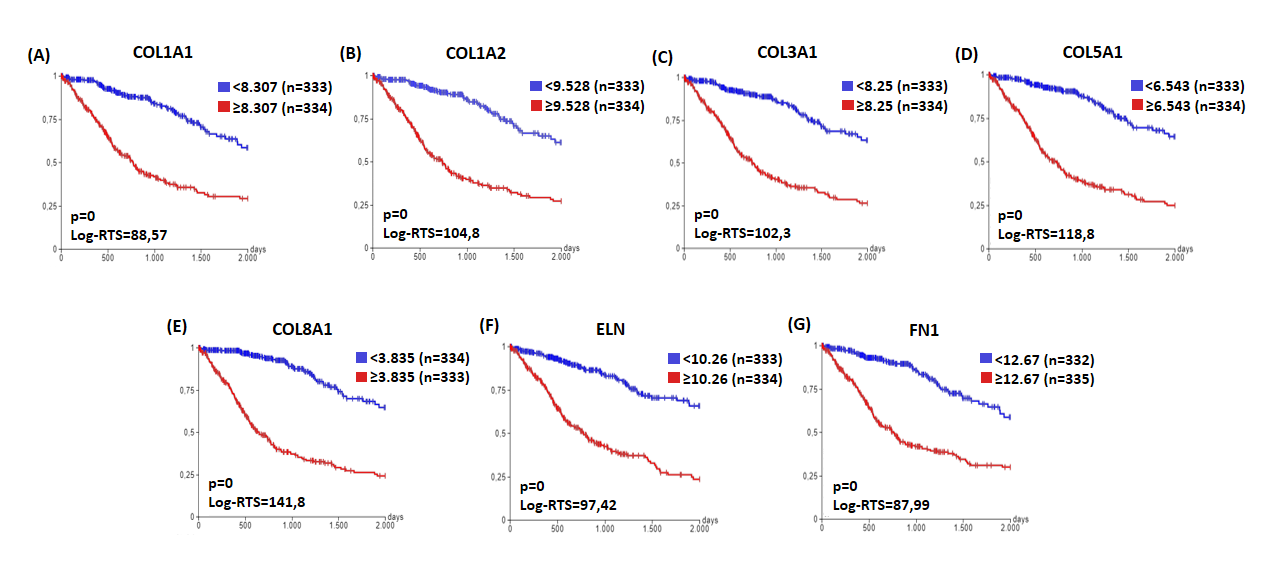

Supplement: Supplementary file 2 — Figure 2 [file CAM4-12-3830-s001.tif]
