# Supplementary material for: Identification of thioredoxin domain containing family members' expression pattern and prognostic value in diffuse gliomas via in silico analysis
Source: Cancer Med. 2022 Sep 15;12(3):3830–44. doi: 10.1002/cam4.5169 (PMC9939227; doi:10.1002/cam4.5169)
Supplement: Supplementary file 3 — Appendix S1 [file CAM4-12-3830-s002.docx]

**Gene expression analysis based on vital status**

Patients with primary tumor in the TCGA-GBMLGG dataset were analyzed using the UCSC Xena database (<https://xena.ucsc.edu/>)[18]. Only those cases having RNA-seq data were included. Living and deceased patients were divided in two groups and the relative TXNDC5 expression level in each group was plotted. Transcript levels were calculated by log2(norm_count+1) in which norm_count refers to the RSEM normalized count.
